# E-learning in Saudi Arabia: ‘To E or not to E, that is the question’

**DOI:** 10.4103/1319-1683.74333

**Published:** 2010

**Authors:** Ali M. Al-Shehri

**Affiliations:** *Department of Public Health, King Saud Bin Abdulaziz University for Health Sciences, Riyadh, Kingdom of Saudi Arabia*

**Keywords:** Challenges and development of E-learning in Saudi Arabia, E-learning, vision and strategic planning of E-learning

## Abstract

**Background:**

The Kingdom of Saudi Arabia (KSA) has witnessed unprecedented growth in higher education and E-learning in recent times. In the last five years, one university and five colleges have been commissioned every month; 800 scholarships have been awarded every month for overseas study; a national center for E-learning has been established; and E-units or departments have been set-up in almost every university. E-learning has become important for discussion to quote Shakespeare ‘To E or not to E that is the question.’

**Objectives:**

To examine current and future developments and challenges of E-learning in KSA.

**Materials and Methods:**

A qualitative approach was used to explore views of 30 senior academicians involved in E-learning during their attendance at a two-week course on the subject.

**Results:**

All participants considered themselves as decision makers on E-learning in their units or departments. They felt that E-learning had come to stay, but acknowledged challenges in respect of resources, organization, management, and information technology.

**Conclusion:**

The fast development of E-learning poses many challenges. Clear vision and strategic planning with prospective E-learners in mind are essential to make E-learning programs cost effective.

## INTRODUCTION

*‘To be, or not to be: that is the question’* -

*William Shakespeare (Hamlet)*

The market for E-learning is estimated as more than 53 billion US dollars. Although the largest market is in USA, the strongest growth is in Asia.[1] Saudi Arabia, as the largest market and economy in the Middle East, has of late witnessed huge expansion in higher education and E-learning. Over the last five years, the growth in higher education has been the opening of one university every three months; five colleges every month; 800 scholarships being awarded to students to study abroad every month; and an extension of higher education from 15 to 86 districts.[2] The move toward E-learning seems to be fast and strong. There have been a number of initiatives to introduce E-learning to Kingdom of Saudi Arabia (KSA). These include orientation sessions and campaigns on the subject; short and long courses for interested participants; establishment of E-learning units in universities and educational organizations; establishment of National Center for E-learning; and the launching of local E-learning programs that aim at national certification for E-learning.

This article shares views and interpretations of E-learning in KSA, drawing on literature and information gathered from 30 academicians and decision makers from all universities and the National Center of E-learning in KSA. The aim is to explore ideas on current and future developments of E-learning in KSA from the decision makers. Findings and interpretations of this study may be helpful in developing organizational vision[3] and strategic planning for E-learning in KSA.

## MATERIALS AND METHODS

Together with 30 participants from all universities of SA and the National Center, the author attended a two-week course entitled ‘E-Learning and the Future of Digital Education’ at The University of Manchester. All participants had knowledge and experience in E-learning and were considered decision makers in their respective organizations. In fact, the course was meant to ‘Train the Trainers’ in E-learning and was sponsored by Ministry of Higher Education. Details of the course can be obtained from National Center for E-learning and distance education.

The learning environment of the course was used to explore the views of the participants on E-learning in the form of one-to-one dialogue or group discussion during small-group tasks during tea and coffee breaks, over lunch and dinner at social gatherings, and other activities. These informal discussions were used to gather information from participants without making them aware of the study. This was in order to minimize the effects of research on participants’ opinion. However, at the end of the course, participants’ permission was taken to use their views and comments in this article on E-learning in KSA.

‘Chats and Dialogues’ with participants focused on the following questions: How do you describe yourself in relation to E-learning in your unit or organization: decision maker or user or other? How do you see E-learning now and in the future of your unit or organization, in particular, and in Saudi Arabia in general? What challenges do you face now and what others do you expect in the future? No notes were made during the chats, but the main ideas were written down immediately afterwards.

This approach gave flexibility to both participants and researcher to tackle the issues from different perspectives and facilitate free reflection on the whole subject.[4] Concepts, rather than literal statements or opinions, were elicited from participants. For example, if a participant mentioned ‘bureaucracy’ in response to the question on the challenges faced in the unit, it was interpreted as a concept of organization and management as shown below. The response ‘money’ or ‘lack of trained staff’ was categorized as resources. The study tried to make use of anecdotes,[5] feelings, judgments, expectations, and views of those senior and experienced participants to get a clear vision of E-learning in KSA. In other words, a qualitative approach was used for data gathering: ‘Qualitative researchers try to interact with their subjects in a natural, inobtrusive, and nonthreatening manner.’[6]

## RESULTS

All participants described themselves as decision makers in relation to E-learning in their units or organizations, which their titles and backgrounds confirmed. Responses to ‘How do you see E-learning in your unit/organization’ ranged from ‘still at the beginning’ to ‘We are well advanced in E-learning.’ However, it was clear that the majority saw themselves as being in an advanced stage. Almost all saw E-learning as an inevitable development in KSA and were most optimistic about its future. Challenges mentioned were related to resources (human, materials, and financial); organization and management (reporting relationships, links and authority, freelance *vs* controlled administration); technical and infrastructure (availability of enough technical support; capacity and coverage of telecommunication). Some felt that budgetary allocation to E-learning was the greatest challenge, whereas others felt the biggest challenge was the knowledge and skills of teachers and learners. Still others felt that the greatest challenge was the infrastructure for technology and telecommunications. Another group felt that organizational relationship of all those involved in E-learning posed the greatest challenge. Despite these challenges, almost all agreed that E-learning was necessary for KSA. These challenges are discussed below in the light of literature on E-learning.

## DISCUSSION

Despite the recent impressive development and expansion of higher education in KSA, there are still not enough places in the universities to accommodate the large number of applicants and meet the growth of the population. Almost half of the population of KSA is at or under college age.[7] Every academic year, thousands of students are left without a place at university to study their subject of choice. Although many universities have recently been opened, the demand for place is still much more than is available. This is compounded by the fact that KSA is a large country with issues of accessibility, particularly for females and those who cannot travel to the main cities where the universities are located. The reasons above confirm the views of participants in this study that part of the solution to this problem is to be found in E-learning. In fact, the fast pace of development toward E-learning in KSA is impressive, considering the country’s relative youth in learning and education. To use the words of Shakespeare’s Hamlet, it is perhaps appropriate in KSA to say: ‘to E or not to E, that is the question.’

It is necessary to pause and give careful thought to certain issues with far-reaching consequences before embarking on full-scale national-certified E-learning programs. The following are some such issues emerging from the literature, and participant’s views have to be given serious thought by the organizations concerned in KSA.

### Education and technology

‘*Information and Communication Technologies (ICT) evolve at a quick pace and affect the way we live, work, access information, communicate and learn.*’[8] Internet and web technology can assist in transforming education in the 21^st^ century, but there is always a risk of putting technology before education, and the claim is that E-learning enables learners to be more self-directed, inquisitive, and reflective. However, the desire to master technology may observe this ideal. During the ‘chats’ with participants as well as the actual course, it was clear that most of the priority was to master the technology. Participants were more interested in acquiring the technical procedures of how to conduct E-learning rather than finding out the educational principles of learning. This was undoubtedly expected because participants look for their learning needs rather than giving priority to technology over education. However, most of participants lost interest when instructors talked about learning theories and reflective practice. However, it is worth remembering that ‘*there is a common misconception that e-learning is mainly about technology … technology is not what learning is all about*.’[8] Learning is essentially about change in attitudes, skills, and knowledge.

### Inequity and variations

‘*E-learning (or sometimes Electronic Learning or eLearning) is a term which is used a great deal but with diverse definitions … it has different meanings to different people*.’[1] In this study, though most of participants felt that their units or organizations were advanced in E-learning, the meanings they adducted to E-learning varied and some saw it as a web-based distance education, whereas others saw it as technology-mediated learning. Digital zing classrooms are not what E-learning is about. However, it was clear from the discussions with participants that some were really advanced in their understanding of E-learning but lacked structural support, whereas others who were not so advanced had great support from other units or organizations. With better organization and management, these inequities and variations can be utilized as strengths rather than weaknesses.

### Organization and management

During the course, we learned about many developments of E-learning in different universities and institutions. There was no clear organizational links or reports to ensure coordination and collaboration among different bodies involved in E-learning. The existence of National Center of E-learning came as a surprise to the majority of participants. The fact that it had no official oversight of the development of E-learning in different universities and organization may explain, at least partly, this lack of awareness. Giving freedom to different organization to adopt E-learning in their own way may present opportunities for more innovations and independence.

However, there is the risk of waste of resources and disintegration of the whole process of E-learning. With proper organization and management, technical support and infrastructure required by different organizations would be easier to identify and thereby improve the efficiency and effectiveness of the pursuit of the national objective appropriate in E-learning in KSA.

### Technical support and infrastructure

Almost all participants expressed concerns about the availability of enough technical support and the capacity of infrastructure to cope with the potential size of E-learning. Supporting flow and processing of information in terms of hardware, software, policies and procedures, and capacity to deal with problems that arise are major challenges for developed countries[9,10] that let alone developing countries. E-learning software applications such as learning management system, managed learning environment, and others are flourishing and need proper hardware infrastructure and technical support. This support should be available in all regions and not limited to major cities. Judging with the current problems on telecommunication coverage of the whole country, any moves that embark large-scale E-learning projects should be made with caution.

In addition, the question of ‘to E or not to E’ in Saudi Arabia cannot be addressed properly unless the following two other issues are tackled: (1) Developing organizational vision and strategic planning for E-learning and (2) Identifying E-learners.

### Organizational vision and strategic planning

The following organizations are a few of many involved directly or indirectly with E-learning in KSA: Ministry of Higher Education, National Center of E-learning, universities, Ministry of Telecommunication, King Abdulaziz City for Science and Technology, educational and training institutions, and the private sector (educational and business firms). These organizations as stakeholders need to come together to formulate a collective vision for E-learning for the whole country. This vision should give some direction with the present realities through risks and opportunities into the future.[3] A common vision for all stakeholders means a common purpose and clear direction to the future. Thus, strategic planning for E-learning in SA would consider the present realities of each organization, their risks and opportunities and attainable goals within a time frame.[3] The development of this vision depends on identifying the customers, that is, the E-learners.

### Identifying our E-learners

‘*E-LEARNERS FAIL! Not one or two, here and there, but large numbers of them. Some studies suggest more than half of would-be e-learners either never take advantage of e-learning possibilities or never finish their first program*.’[9] This quotation is the opening sentence of the book[9] that emphasizes the importance of preparing E-learners before embarking on major e-learning programs. We need to know the characteristics, motivations, and potential of E-learners before asking them to join E-learning programs. Traditional classroom learning programs have been the norm for years and to ask learners not to come to class may feel strange and perhaps difficult to accept. More importantly, E-learning might require different skills, knowledge, and attitudes.[9,10]

Therefore, there is an urgent need to do major studies on the prospective E-learners to find out what their perception of E-learning is, what skills they have, and what they understand. It is vital to find out what is motivating them to participate in the E-learning programs. It would be necessary to find out where, when, and how they want to E-learn. There are many other questions that must be resolved and the results of which will provide useful information to determine the modalities for implementing cost-effective major E-learning programs.

### Limitations of the study

Reliance on informal chatting and dialogues without recording or transcribing what was actually said is a clear limitation. There is the risk of the loss of some data and compromise of validity. However, issues of concerns and challenges mentioned by participants were similar to those reported in literature.[8–10] Certainly, capturing actual statements or comments of participants would substantiate qualitative results, but would not alter the outcome validity of the study that E-learning in Saudi Arabia is flourishing, poses many challenges, requires strategic planning based on clear vision, and demands an appraisal of the E-learners.

Another limitation of the study is the limited target of number of senior academicians and e-learning tutors. This purposeful sampling is acceptable in qualitative research. Participants were considered a focus group chosen because of their experience, insights, and knowledge of e-learning. Their views carry a significant weight that should be acknowledged in strategic planning of E-learning in KSA. Moreover, the findings and limitations of this study demand further research (both qualitative and quantitative) on E-learning.
